# Supplementary material for: Climatic drivers of Verticillium dahliae occurrence in Mediterranean olive-growing areas of southern Spain
Source: PLoS One. 2020 Dec 30;15(12):e0232648. doi: 10.1371/journal.pone.0232648 (PMC7773261; doi:10.1371/journal.pone.0232648)
Supplement: S1 Table — Each pairwise interaction was separately included in the top-ranked model for V. dahliae occurrence (see Table 1 in the main manuscript). We ordered the models by their AIC values and checked whether the interaction term was significant or not. (DOCX) [file pone.0232648.s001.docx]

**S1 Table**. Comparison of model fit when including interaction terms between climatic variables and covariates. Each pairwise interaction was separately included in the top-ranked model for *V. dahliae* occurrence (see Table 1 in the main manuscript). We ordered the models by their AIC values and checked whether the interaction term was significant or not.

| Interaction term | p-value | AIC |
| --- | --- | --- |
| Isothermality x Watering | 0.04 | 559.8 |
| RainfallSeasonality x Watering | n.s. | 562.41 |
| Isothermality x Plant material origin | n.s. | 563.79 |
| RainfallSeasonality x Plant material origin | n.s. | 563.9 |

*n.s.: no significant at 0.05*
