# Supplementary material for: Climatic drivers of Verticillium dahliae occurrence in Mediterranean olive-growing areas of southern Spain
Source: PLoS One. 2020 Dec 30;15(12):e0232648. doi: 10.1371/journal.pone.0232648 (PMC7773261; doi:10.1371/journal.pone.0232648)
Supplement: S2 Table — The performance of the models was evaluated using the Log‐Loss. Lower values of this model selection index indicate better model performance. Performance measures were averaged throughout the ten data partitions. Improvement was calculated as [(Top-ranked—Only-intercept) / Only-intercept] * 100. Each data partition was made by randomly splitting the data into 10 distinct blocks and using 9 to train and 1 to test. (DOCX) [file pone.0232648.s002.docx]

**S2 Table**. Comparison between the top-ranked model and the intercept-only model using 10-fold cross-validation. The performance of the models was evaluated using the Log‐Loss. Lower values of this model selection index indicate better model performance. Performance measures were averaged throughout the ten data partitions. Improvement was calculated as [(Top-ranked - Only-intercept) / Only-intercept] * 100. Each data partition was made by randomly splitting the data into 10 distinct blocks and using 9 to train and 1 to test.

| Partition | Only-intercept | Top-ranked | Improvement |
| --- | --- | --- | --- |
| 1 | 0.532 | 0.396 | -34.286 |
| 2 | 0.433 | 0.383 | -12.941 |
| 3 | 0.444 | 0.364 | -22.007 |
| 4 | 0.419 | 0.302 | -38.586 |
| 5 | 0.454 | 0.318 | -42.713 |
| 6 | 0.417 | 0.362 | -15.166 |
| 7 | 0.433 | 0.367 | -17.821 |
| 8 | 0.371 | 0.364 | -1.800 |
| 9 | 0.448 | 0.383 | -16.902 |
| 10 | 0.390 | 0.324 | -20.131 |
| Mean | 0.434 | 0.357 | -22.235 |
| SD | 0.043 | 0.031 | 12.642 |
