# Supplementary material for: Climatic drivers of Verticillium dahliae occurrence in Mediterranean olive-growing areas of southern Spain
Source: PLoS One. 2020 Dec 30;15(12):e0232648. doi: 10.1371/journal.pone.0232648 (PMC7773261; doi:10.1371/journal.pone.0232648)

**S1 Fig.** Map of WorldClim climatic variables averaged for the years 1970-2000 in Granada province (southern Spain).


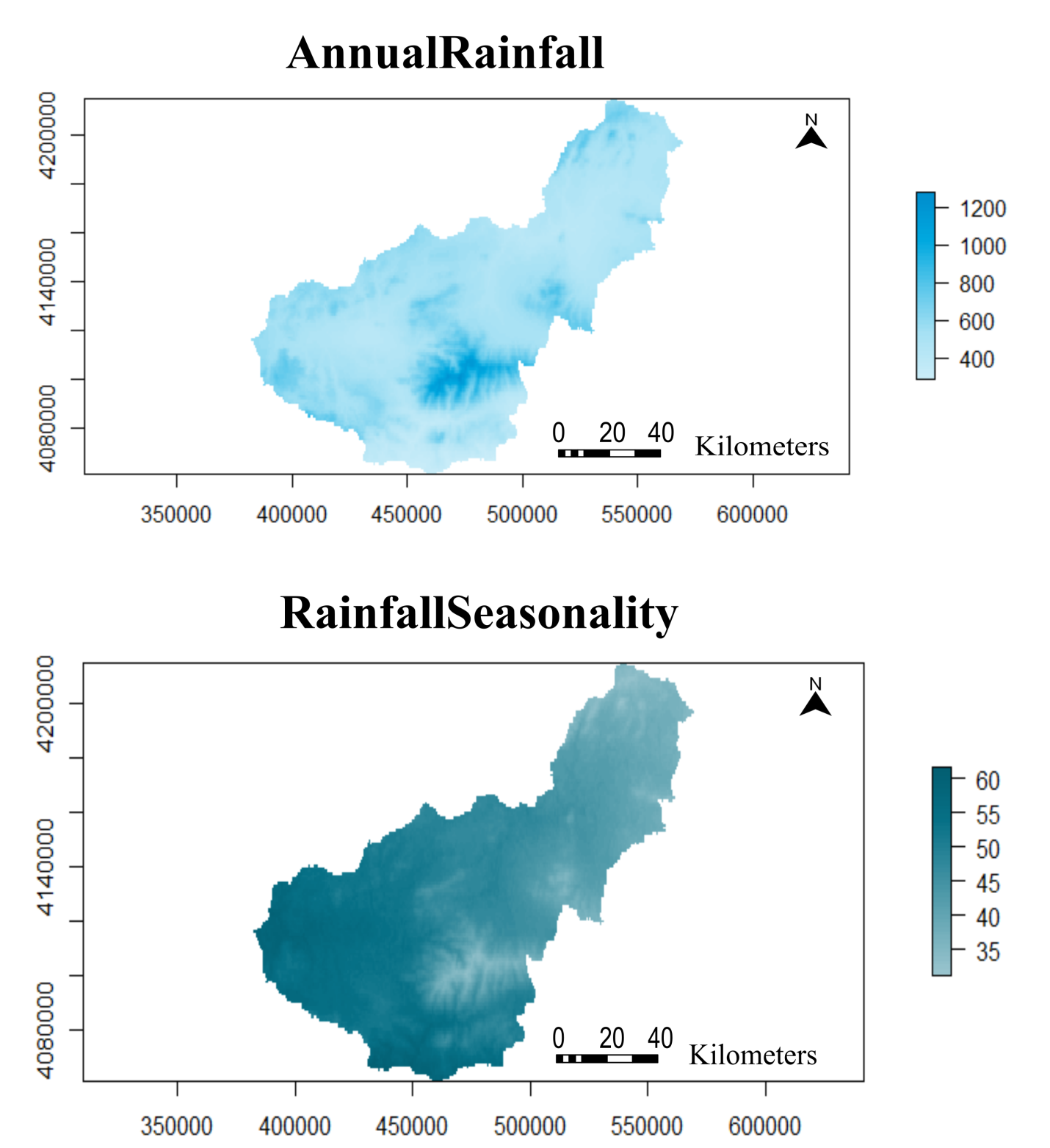


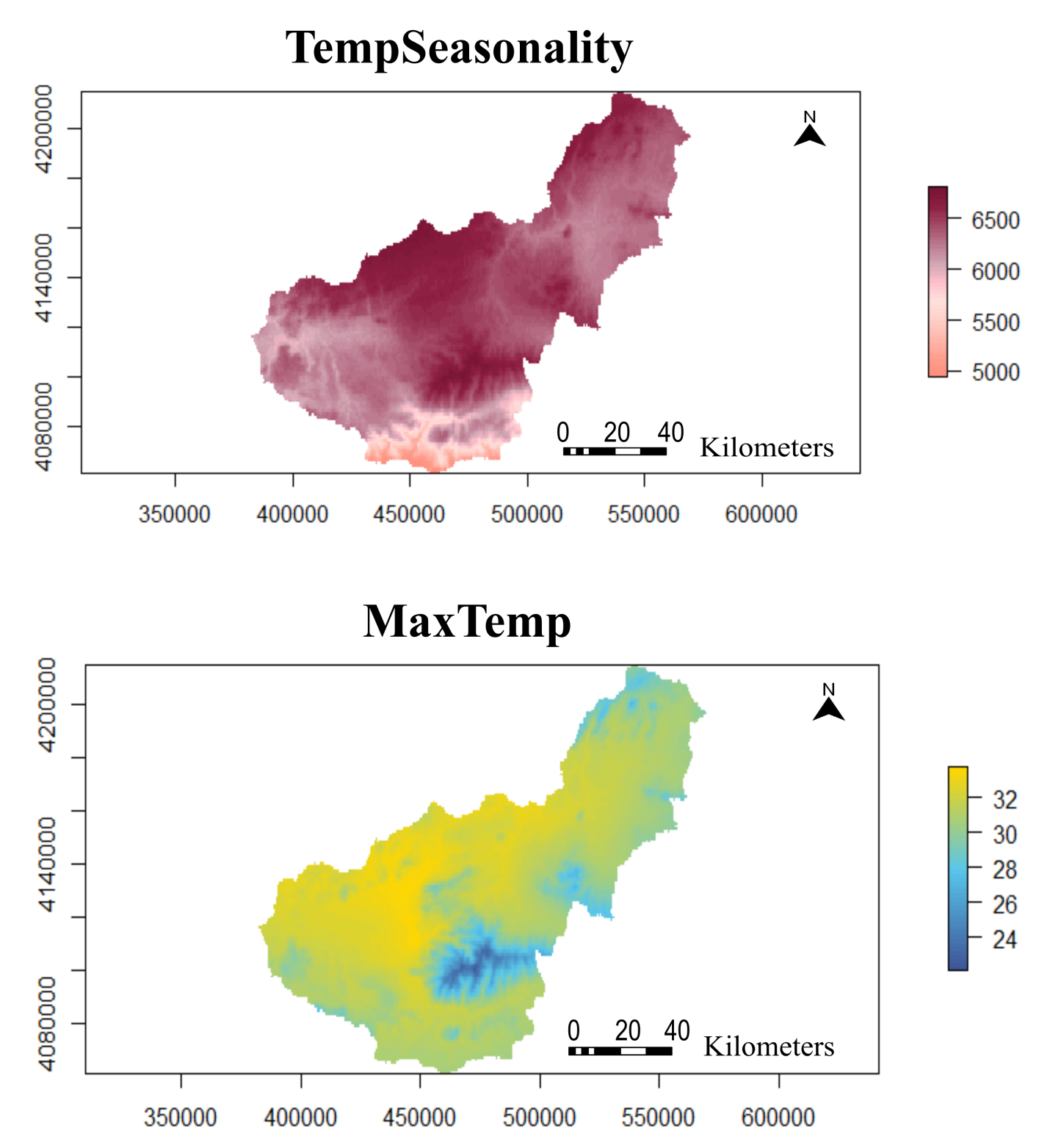


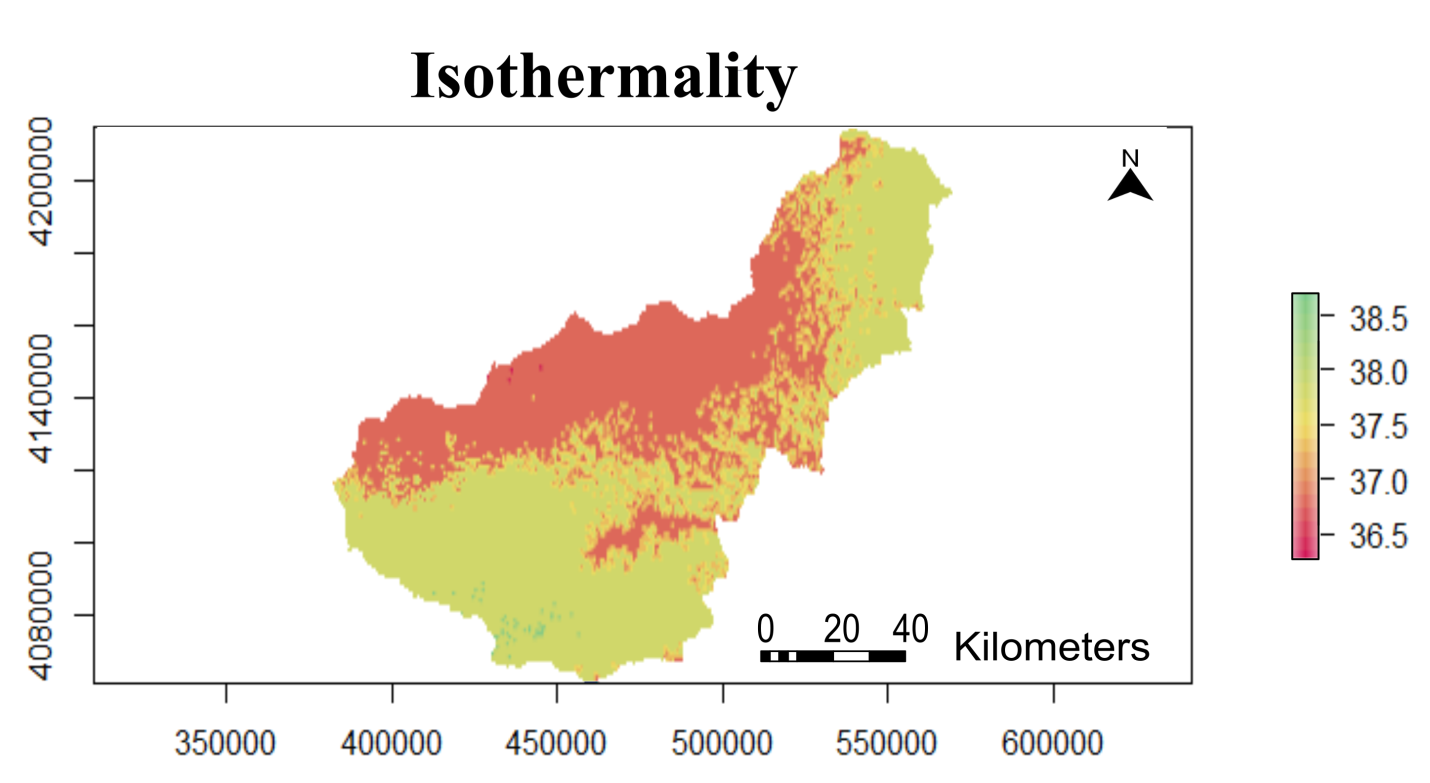

Supplement: S1 Fig — (DOCX) [file pone.0232648.s003.docx]
