# Supplementary material for: Climatic drivers of Verticillium dahliae occurrence in Mediterranean olive-growing areas of southern Spain
Source: PLoS One. 2020 Dec 30;15(12):e0232648. doi: 10.1371/journal.pone.0232648 (PMC7773261; doi:10.1371/journal.pone.0232648)
Supplement: S2 Fig — (a) Bubble plot of the standardized residuals from the top-ranked model (see Table 1 in the main manuscript) including an interaction term between isothermality and watering. (b) Spline correlogram of the residuals using the function spline.correlog in the “ncf” R package [1]. The spatial dependence is tested as a continuous function of distance. The gray shadows represent the 95% confidence interval. (DOCX) [file pone.0232648.s004.docx]

**S2 Fig.** Analysis of the top-ranked model residuals. (a) Bubble plot of the standardized residuals from the top-ranked model (see Table 1 in the main manuscript) including an interaction term between isothermality and watering. (b) Spline correlogram of the residuals using the function *spline.correlog* in the “*ncf”* R package [1]. The spatial dependence is tested as a continuous function of distance. The gray shadows represent the 95 % confidence interval.


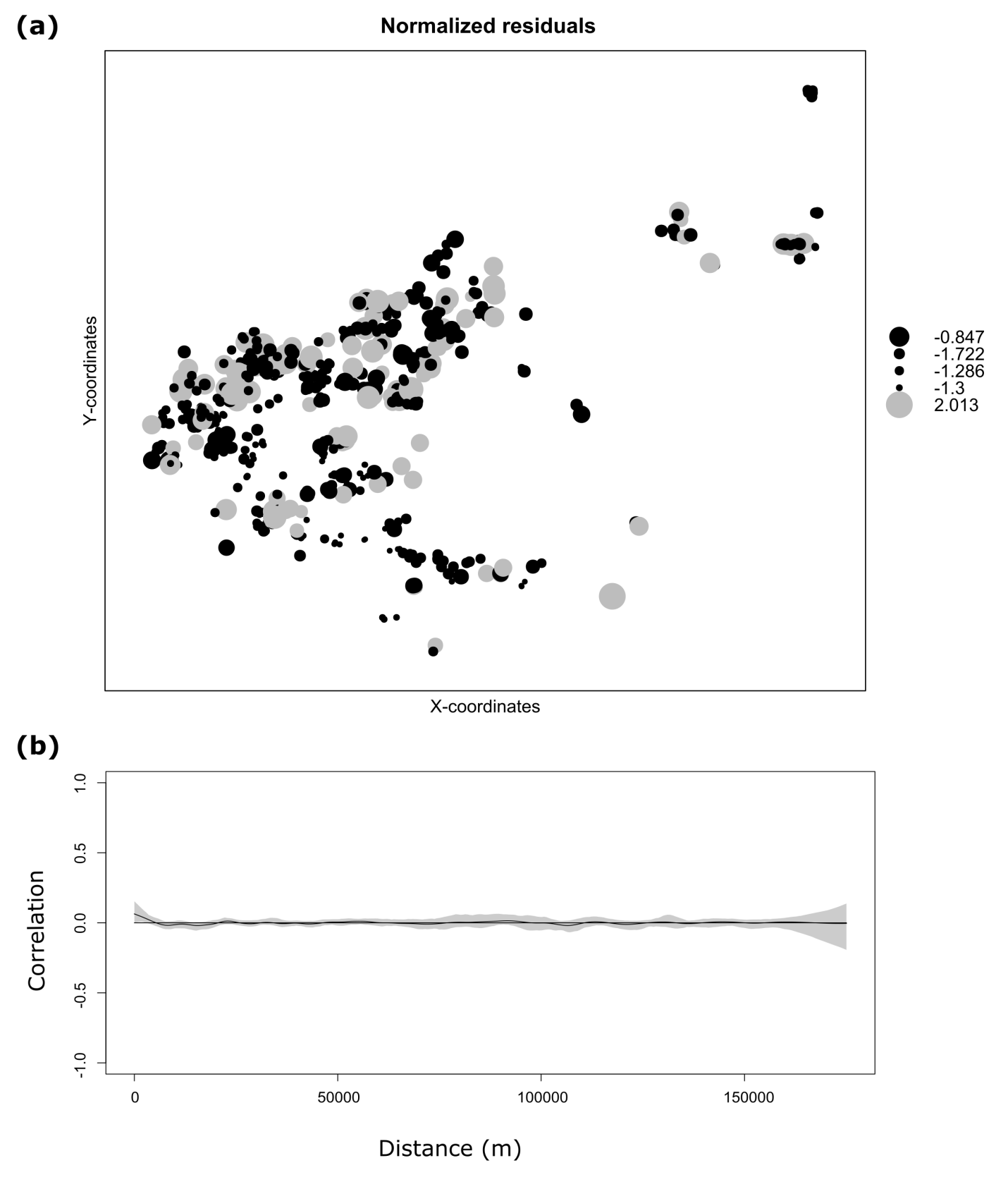


**Reference**

1. Bjornstad ON. ncf: Spatial Covariance Functions. R package version 1.2-8. 2019. Available from: https://CRAN.R-project.org/package=ncf
