# Supplementary material for: Climatic drivers of Verticillium dahliae occurrence in Mediterranean olive-growing areas of southern Spain
Source: PLoS One. 2020 Dec 30;15(12):e0232648. doi: 10.1371/journal.pone.0232648 (PMC7773261; doi:10.1371/journal.pone.0232648)
Supplement: S1 Appendix — (DOCX) [file pone.0232648.s005.docx]

**S1 Appendix**. Comparison between boosted regression trees and binomial models for *V. dahliae* occurrence.

We fitted boosted regression trees (BRT) for *V. dahliae* occurrence and compared their predictive performance with the one yielded by the top-ranked binomial model (see Table 1 in the main manuscript) using a 10-fold cross-validation. We followed the recommendations provided by Elith et al. (2008) to find the parameters combination that better predictions yields while avoids overfitting. To do that, we set different values for tree complexity (i.e., the number of nodes in a tree), learning rate (i.e., the weight applied to individual trees), and bag fraction (i.e., the proportion of observations used in selecting variables). We assessed predictive performance by calculating the Log‐Loss across the data partitions. S1 Table shows models’ performance when the bag fraction was set to 0.75. A bag fraction of 0.5 yielded very similar results as the ones shown below, therefore they are omitted here. The R code to reproduce the model comparisons is available in https://github.com/jmrmcode/Verticillium-wilt-Dataset.

**S3 Table**. Comparison between BRT and binomial models. The performance of the models was evaluated using the Log-Loss. Lower values of Log-Loss indicate better model performance. Performance measures were averaged throughout the data partitions. The “Ratio” column shows the number of BRT models (numerator) that performed better than the binomial models (denominator) for each combination of parameters.

| BRT | | | Binomial |  |
| --- | --- | --- | --- | --- |
| Tree complexity | Learning rate | Log-Loss ±SE | Log-Loss ±SE | Ratio |
| 1 | 0.01 | 0.360 ±0.023 | 0.353 ±0.019 | 3/7 |
|  | 0.005 | 0.366 ±0.027 | 0.359 ±0.023 | 4/6 |
|  | 0.001 | 0.364 ±0.028 | 0.357 ±0.028 | 3/7 |
| 5 | 0.01 | 0.357 ±0.011 | 0.358 ±0.009 | 4/6 |
|  | 0.005 | 0.350 ±0.026 | 0.354 ±0.028 | 5/5 |
|  | 0.001 | 0.368 ±0.014 | 0.362 ±0.016 | 4/6 |
| 10 | 0.01 | 0.362 ±0.021 | 0.361 ±0.025 | 6/4 |
|  | 0.005 | 0.360 ±0.019 | 0.356 ±0.019 | 3/7 |
|  | 0.001 | 0.366 ±0.020 | 0.360 ±0.021 | 4/6 |

The differences between both modeling techniques were negligible although, in most of the cases, the binomial regression models yielded slightly better performance than that of the BRT models. These results support the generalized linear models adopted in our study.

**References**

Elith J, Leathwick JR, Hastie T. A working guide to boosted regression trees. J Anim Ecol. 2008;77: 802-813. doi: https://doi.org/10.1111/j.1365-2656.2008.01390.x
