# Supplementary material for: Climatic drivers of Verticillium dahliae occurrence in Mediterranean olive-growing areas of southern Spain
Source: PLoS One. 2020 Dec 30;15(12):e0232648. doi: 10.1371/journal.pone.0232648 (PMC7773261; doi:10.1371/journal.pone.0232648)
Supplement: Attachment — Submitted filename: Response to Reviewers.docx [file pone.0232648.s006.docx]

***General comments to reviewers and Academic Editor***

Dear Mr. Di Febbraro:

Thank you for the opportunity to revise our manuscript, “Climatic drivers of *Verticillium dahliae* occurrence in Mediterranean olive-growing areas of southern Spain” to *PLOS ONE*. In our response below, we include an overview of our revisions as well as our point-by-point responses to the reviewers. In the point-by-point responses, we have italicized the reviewers’ comments. We look forward to your decision on the revised manuscript.

**Overview of revisions**

The two reviewers are positive about the manuscript; Referee #1 highlights that our study brings timely insights that help to set appropriate irrigation management in order to reduce the propagation of the pathogen. Referee #2 provides really valuable suggestions regarding methodological aspects and the discussion of management implications derived from our results. Consequently, we focused our revisions on improving the clarity of the manuscript, following reviewer recommendations. We have fitted boosted regression trees for *V.dahliae* occurrence data and assessed their performance. We have revalidated our models using the standard 10-fold cross-validation method and provided new figures and tables in support of our conclusions. We have also added text to the discussion section to better connect our results with their implications in irrigation management.

**JOURNAL REPORT(S):**

*1. Please ensure that your manuscript meets PLOS ONE's style requirements, including those for file naming.*

We have checked PLOS ONE’s style requirements and now the manuscript meets them.

*2. We note that you have stated that you will provide repository information for your data at acceptance. Should your manuscript be accepted for publication, we will hold it until you provide the relevant accession numbers or DOIs necessary to access your data.*

The data set and metadata information can be accessed at the following [repository](https://github.com/jmrmcode/Verticillium-wilt-Dataset). Both link and DOI (10.5281/zenodo.4004906) are provided on lines 159-160 in the main manuscript.

*3. We note that Figure 1 in your submission contain map images which may be copyrighted. All PLOS content is published under the Creative Commons Attribution License (CC BY 4.0), which means that the manuscript, images, and Supporting Information files will be freely available online, and any third party is permitted to access, download, copy, distribute, and use these materials in any way, even commercially, with proper attribution. For these reasons, we cannot publish previously copyrighted maps or satellite images created using proprietary data, such as Google software (Google Maps, Street View, and Earth).*

Thank you for noting us about it. Since the base map used in the old version of Fig 1 did not add any indispensable information, we have removed it from the current version, replaced it with a white background, and updated the caption (see lines 115-122). All the data shown in the updated version of Fig 1 is compatible with the CC-BY 4.0 license (as we now claim in the caption). Please, check the following links showing the terms of use. [*V. dahliae* occurrence data](https://github.com/jmrmcode/Verticillium-wilt-Dataset), the [administrative boundaries](http://www.juntadeandalucia.es/medioambiente/site/portalweb/menuitem.30d4b35a97db5c61716f2b105510e1ca/?vgnextoid=f910b3a5172f5310VgnVCM2000000624e50aRCRD&vgnextchannel=f078193566a68210VgnVCM10000055011eacRCRD&lr=lang_es) of Granada province, and the [digital elevation model](https://www.juntadeandalucia.es/institutodeestadisticaycartografia/ieagen/avisoLegal/index.htm#cc).

**REFEREE REPORT(S):**

**Reviewer: 1**

Comments to the Author

*In this study I appreciated the attempt to investigate the correlation between climatic variables and the probability of occurrence V. dahliae pathogen by applying modelling and statistic approaches. Moreover, the results of your study may be very helpful to provide information about how irrigation could be better managed in order to modulate the effect of climate on fungus’s thrive and reduce the risk of pathogen’s spread.
Nevertheless, before I recommend this paper for publication, the authors should address some issues that I detected.*

Response: We thank the reviewer for their positive feedback.

*Introduction
1.1	Line 47: I would write “in Andalusia (southern Spain) which is the world’s leading olive tree grower…”*

Response: Done. Please see lines 46-49.

*Methods
1.2	Line 139: A citation about the application of the Spearman rank-correlation is needed.*

Response: We have added the following reference in support of the Spearman rank-correlation. See line 144 in the manuscript. In addition, please see “The global model” subsection in Gallien et al. (2012) where the authors used a correlation value of 0.6 as a threshold to discard variables.

Gallien L, Douzet R, Pratte S, Zimmermann NE, Thuiller W. Invasive species distribution models – how violating the equilibrium assumption can create new insights. Glob. Ecol. Biogeogr. 2012; 21(11): 1126-1136 doi: <https://doi.org/10.1111/j.1466-8238.2012.00768.x>

*1.3	Line 159: There are other studies that have investigated how climatic factors might affect the distribution of plant pathogenic fungi, e.g.: Bosso, L., Luchi, N., Maresi, G., Cristinzio, G., Smeraldo, S., & Russo, D. (2017). Predicting current and future disease outbreaks of Diplodia sapinea shoot blight in Italy: species distribution models as a tool for forest management planning.*

Response: We thank the reviewer for pointing us in the direction of this interesting reference. We have added it, along with the following references, to the current version of the manuscript.

References:

Desprez-Loustau, ML, Robin C, Reynaud, G, Déqué M, Badeau, V, Piou, D, et al. Simulating the effects of a climate-change scenario on the geographical range and activity of forest-pathogenic fungi. Canadian J Plant Pathol. 2007;29(2): 101-120. doi: https://doi.org/10.1080/07060660709507447

Bosso L, Luchi N, Maresi G, Cristinzio G, Smeraldo S, Russo D. Predicting current and future disease outbreaks of *Diplodia sapinea* shoot blight in Italy: species distribution models as a tool for forest management planning. Forest Ecol. Manag. 2017;400: 655-664. doi:10.1016/j.foreco.2017.06.044

Doohan FM, Brennan J, Cooke BM. Influence of climatic factors on Fusarium species pathogenic to cereals. In: Xu X, Bailey JA, Cooke BM eds. Epidemiology of Mycotoxin Producing Fungi. Springer, Dordrecht. 2003. pp. 755-768. doi: <https://doi.org/10.1007/978-94-017-1452-5_10>

Launay M, Caubel J, Bourgeois G, Huard F, Garcia de Cortazar-Atauri I, Bancal MO, Brisson N. Climatic indicators for crop infection risk: Application to climate change impacts on five major foliar fungal diseases in Northern France. Agr Ecosyst Environ. 2014;197: 147-158. doi: https://doi.org/10.1016/j.agee.2014.07.020

Please see line 165.

*1.4	Line 186: Please, clarify if you have used the Log Loss with presence-only test data.*

Response: We appreciate the comment and have rephrased the sentence as follows:

“We assessed how accurate the predictions of the model were by comparing the Log‐Loss achieved by the null-model (i.e., intercept-only) as a baseline with that of the top-ranked model using a 10-fold cross-validation of the presence-absence sampled data”. See lines 191-193.

*1.5	Line 198 and line 212: Please, remove citations from results session and eventually move them to discussion session.*

Response: We agree and appreciate the comment. We have removed reference [54] in support of the AIC scores criterion since it was earlier included in the “Materials and Methods” section (see lines 184-185). In addition, we have moved references [14] and [33] in support of the marginal effects of the covariates to the “Discussion” section. The text is as follows:

Reference [14] is used in lines 272-273:

“We also corroborated previous results that indicated irrigation was a significant abiotic factor for *V. dahliae* [14], since there was a strong positive correlation between irrigated olive groves and *V. dahliae* occurrence”.

Reference [33] is used in lines 286-288:

“Regarding the marginal effect of the plant material origin, our results were also consistent with that found by [33] in which young plants from nurseries were found to increase the probability of *V. dahliae* occurrence.”

References used in response:

Burnham KP, Anderson DR. Model selection and multimodel inference. A practical information-theoretic approach. New York: Springer-Verlag. 2002

Rodríguez E, García-Garrido JM, García PA, Campos M. Large-scale epidemiological study and spatial patterns of Verticillium wilt in olive orchards in southern Spain. Crop Prot. 2009;28: 46-52. doi: https://doi.org/10.1016/j.cropro.2008.08.009

Rodríguez E, García-Garrido JM, García PA, Campos M. Implications of plant material origin, land use history and soil properties in the incidence of Verticillium wilt in olive groves. J Plant Pathol. 2011;93(1): 111-118. doi: http://dx.doi.org/10.4454/jpp.v93i1.280

*1.6	Lines 228-229: Authors should show the full results of log loss evaluation in supplementary materials.*

Response: We totally agree and have added such information in S2 Table (line 238). Please note that we have updated the Log-Loss values according to Referee #2's comment 2.8 (see below).

*Discussion
1.7	Lines 236-237: Authors should specify why they use the term “sophisticated” and “primary” for the different variables.*

Response: We thank the Reviewer for this comment and the opportunity to explain better the use of these two terms. We have added the following paragraph to the “Discussion” section to clarify what “sophisticated” and “primary” climatic factors refer to.

“Here, “sophisticated” climatic factors refer to climatic variables that integrate different aspects of climate components at multiple temporal scales. For example, isothermality represents the ratio between temperature oscillations at two temporal scales: daily and seasonal, and it captures wider ecological trends better than the temperature for a given day (i.e., a “primary” variable) due to the inherent variability associated with weather. Isothermality was the most important climatic factor for the pathogen occurrence, suggesting an intricate relationship between *V. dahliae* and climate as discussed below.” See lines 247-254.

*1.8	Line 248: Please, a citation is needed.*

Response: We agree with the reviewer. We have added the following references in support of our statement. See lines 264-267.

Jiménez-Díaz RM, Cirulli M, Bubici G, Jiménez-Gasco LM, Antoniou PP, Tjamos EC. 2012. Verticillium wilt, a major threat to olive production: Current status and future prospects for its management. Plant Dis. 2012;96: 304-329. doi: https://doi.org/10.1094/PDIS-06-11-0496

Pérez-Rodríguez M, Serrano N, Arquero O, Orgaz F, Moral J, López-Escudero FJ. The Effect of Short Irrigation Frequencies on the Development of Verticillium Wilt in the Susceptible Olive Cultivar ‘Picual’ under Field. Plant Dis. 2016;100(9): 1880-1888. doi: <https://doi.org/10.1094/PDIS-09-15-1018-RE>

*1.9	Lines 257-259: Authors say that irrigation mitigate the negative effect of isothermality on V. dahliae occurrence, however, if I have correctly understood, from fig.2 it results that the combination between isothermality and watering showed an increasing negative effect on occurrences. Please, could you better explain this sentence?*

Response: We really appreciate this comment. The old version of Fig 2 showed that the effect of the interaction between isothermality and watering on *V. dahliae* occurrence was -0.55 (p-value = 0.04). Keeping in mind that watering had two levels, i.e., -irrigated- (used as the reference level) and -rainfed-, the mentioned effect means that when olive groves changed from irrigated to rainfed, the effect of isothermality becomes more negative, specifically by 66.8%, expressed as the rate of change in the cloglog scale. In the older version of the manuscript, we stated such an effect in the opposite sense, i.e., when olive gardens changed from rainfed to irrigated (see lines 210-212 in the older version of the manuscript). The latter interpretation is equivalent to the former one as the effect has the same magnitude but opposite sign. However, to avoid a misinterpretation, -rainfed- has been set as the reference level in the new version of the manuscript (see line 229-230) and, therefore, the effect of the interaction is now shown as positive in the new version of Fig 2.

**Reviewer: 2**

Comments to the Author

*2.1	Thank you for the opportunity to review the manuscript titled ‘Climatic drivers of Verticillium dahliae occurrence in Mediterranean olive-growing areas of southern Spain’.* *This manuscript presents a straightforward study, using species distribution modelling techniques to interrogate conditions potentially favourable to the studied organism. The methods used in this paper are simple but corrected executed. However, there is an argument to be made that with such comprehensive data and well-supported knowledge on drivers of occurrence, more nuanced techniques could be used to explore the research question further (e.g. boosted regression trees are especially suited for interrogating covariate interactions – given that interacting conditions are found to be very important, I think you should consider trying such models).*

Response: We really appreciate this comment and the opportunity to more rigorously validate our modeling approach. We have fitted boosted regression trees (BRT) for *V.dahliae* occurrence and assessed model performance using the standard 10-fold CV method, as suggested by Referee #2 (see comment 2.8 below). We followed the recommendations provided by Elith et al. (2008) to find the combination of parameters that achieves minimum predictive error while avoiding overfitting. To do that, we set different values for tree complexity (i.e., the number of nodes in a tree), learning rate (i.e., the weight applied to individual trees), and bag fraction (i.e., the proportion of observations used in selecting variables), and compared the Log-Loss values yield by the BRT models with those achieved by the GLM approach proposed by us. Table 1 shows models’ performance when the bag fraction was set to 0.75. We omit here the ones achieved when using a value of 0.5 as they were very similar as those shown in Table 1.

Table 1. Comparison between BRT and GLM models’ performance. The performance of the models was evaluated using the Log-Loss. Lower values of Log-Loss indicate better model performance. Performance measures were averaged throughout the ten data partitions. The “Ratio” column shows the number of BRT models (numerator) that performed better than the GLM models (denominator) for each combination of parameters.

| BRT | | | GLM |  |
| --- | --- | --- | --- | --- |
| Tree complexity | Learning rate | Log-Loss ±SE | Log-Loss ±SE | Ratio |
| 1 | 0.01 | 0.360 ±0.023 | 0.353 ±0.019 | 3/7 |
|  | 0.005 | 0.366 ±0.027 | 0.359 ±0.023 | 4/6 |
|  | 0.001 | 0.364 ±0.028 | 0.357 ±0.028 | 3/7 |
| 5 | 0.01 | 0.357 ±0.011 | 0.358 ±0.009 | 4/6 |
|  | 0.005 | 0.350 ±0.026 | 0.354 ±0.028 | 5/5 |
|  | 0.001 | 0.368 ±0.014 | 0.362 ±0.016 | 4/6 |
| 10 | 0.01 | 0.362 ±0.021 | 0.361 ±0.025 | 6/4 |
|  | 0.005 | 0.360 ±0.019 | 0.356 ±0.019 | 3/7 |
|  | 0.001 | 0.366 ±0.020 | 0.360 ±0.021 | 4/6 |

The differences between BRT and GLM models’ performance were tiny and, in most of the cases, the GLM approach achieved a slightly better performance. Therefore, we appeal to Ockham’s razor principle that claims “Pluralitas non est ponenda sine necessitate”, which can be interpreted as: “An explanation of the facts should be no more complicated than necessary” or “Among competing hypotheses, favor the simplest one” (Jefferys and Berger 1992). As there is no objective evidence in support of fitting the data using the BRT approach, we have maintained our original modeling approach to address the goals proposed in the study.

Regarding interactions between climate factors and covariates. Understanding model outcomes, in terms of interpreting and identifying the underlying ecological variability and mechanisms, is crucial when the goal is to make inference on the climate factors influencing species occurrence. Regression models are explicitly hypothesis-driven that allow easily interpreting variable contribution and can test correlative ties and interactions between predictor and response variables while quantifying the sources of variation in data (Miller and Franklin 2002). In consequence, we adopted a regression modeling approach for exploring potential interactions between climatic and abiotic factors as shown in Fig 3.

Finally, the comparison between the two modeling techniques can be reproduced in the “Google Colaboratory” platform using [this link](https://colab.research.google.com/drive/1TPd0n0ysNkVXq3PehXqtlvbNwkddmXh3?usp=sharing) or by running the attached R script in a local environment.

References used in response:

Elith J, Leathwick JR, Hastie T. A working guide to boosted regression trees. J Anim Ecol. 2008;77: 802-813. doi: <https://doi.org/10.1111/j.1365-2656.2008.01390.x>

Jefferys WH, Berger JO. Ockham’s Razor and Bayesian Analysis. American Scientist. 1992;80: 64-72.

Miller J, Franklin J. Modeling the distribution of four vegetation alliances using generalized linear models and classification trees with spatial dependence. Ecol Model. 2002;157: 227–247. doi: <https://doi.org/10.1016/S0304-3800(02)00196-5>

*2.2	Furthermore, the study tries to interrogate landscape-scale drivers of species occurrence with broad WorldClim climatic variables. This is difficult, because climate effects are often more important/easily-observed on larger scales – one would imagine that monthly or even weekly temperature and precipitation averages may have a more pronounced effect on this scale. Even better, soil moisture instead of precipitation, and fine-grained terrain relief data, may also prove to be important to the species, based on previous research highlighted in this manuscript. If possible, the authors should consider using these variables instead of WorldClim.*

Response: We really appreciate this comment and the opportunity to review the use of the “landscape-scale” term in our manuscript. “Landscape-scale” is a term commonly used to refer processes that cover a large spatial scale (Turner et al. 2001), but there is no formal agreement on how large a study area should be to used such a term. Regarding this, our study covered 12,531 km^2^ and contained a wide gradient of climatic conditions that were captured by the WorldClim variables as shown in S2 Fig. Other studies focused on species distribution models also used these variables in similar or even smaller study areas than the one of Granada province (see for example Williams et al. 2009 (1,200 km^2^) in “Diversity and Distributions”; Buckland et al. 2014 (2,040 km^2^) in “PLOS ONE”; Thapa et al. 2018 (13,762 km^2^) in “PLOS ONE”; Jiménez-Valverde et al. 2009 (6,576.4 km^2^) in “Community Ecology”; Chang et al. 2012 (9,206 km^2^) in “PLOS ONE”). Therefore, we think that the use of the WorldClim variables is justified in this case as our study area is large enough. However, since the use of the expression “landscape-scale” can induce confusion, we have replaced it with “large-scale” throughout the new version of the manuscript.

In addition, we agree with Referee #2 that both soil moisture and slope are important drivers for *V. dahliae* occurrence, as it has previously shown in several studies (e.g. López-Escudero et al. 2005; Rodríguez et al., 2011). Nevertheless, corroborating or refuting previous findings is beyond our goals. We are aimed at exploring the climatic sensitivity of the pathogen in semi-arid Mediterranean olive-growing areas, and not at mapping its occurrence; accordingly, our models may not contain all the non-climate factors found to affect *V. dahliae* occurrence. Although “watering” and “plant material origin” are not climatic variables, they were included in our models in order to explore potential interactions with climate as the implications of this may be very important in crop management.

References used in response:

Buckland S, Cole NC, Aguirre-Gutiérrez J, Gallagher LE, Henshaw SM, Besnard A, Tucker RM, Bachraz V, Ruhomaun K, Harris S. Ecological Effects of the Invasive Giant Madagascar Day Gecko on Endemic Mauritian Geckos: Applications of Binomial-Mixture and Species Distribution Models. PLOS ONE 2014;9 e88798. doi: <https://doi.org/10.1371/journal.pone.0088798>

Chang J, Chen D, Ye X, Li S, Liang W, Zhang Z, Li M. Coupling Genetic and Species Distribution Models to Examine the Response of the Hainan Partridge (*Arborophila ardens*) to Late Quaternary Climate. PLOS ONE. 2012:(7) e50286. doi: https://doi.org/10.1371/journal.pone.0050286

Jiménez-Valverde A, Lobo JM, Hortal J. The effect of prevalence and its interaction with sample size on the reliability of species distribution models. Community Ecol. 2009;10:196-205. doi: <https://doi.org/10.1556/ComEc.10.2009.2.9>

López-Escudero FJ, Blanco-López MA. Effects of drip irrigation on population of Verticillium dahliae in olive orchards. J Phytopathol. 2005. 153(4): 238-239. doi: <https://doi.org/10.1111/j.1439-0434.2005.00961.x>

Rodríguez E, García-Garrido JM, García PA, Campos M. Implications of plant material origin, land use history and soil properties in the incidence of Verticillium wilt in olive groves. J Plant Pathol. 2011;93(1): 111-118. doi: http://dx.doi.org/10.4454/jpp.v93i1.280

Thapa S, Chitale V, Rijal SJ, Bisht N, Shrestha BB. Understanding the dynamics in distribution of invasive alien plant species under predicted climate change in Western Himalaya. PLOS ONE 2018;13, e0195752. doi: <https://doi.org/10.1371/journal.pone.0195752>

Turner MG, Gardner RH, O’Neill RV. Landscape Ecology in Theory and Practice: Pattern and Process. Springer-Verlag, New York. 2001 doi: https://doi.org/10.1007/b97434

Williams JN, Seo C, Thorne J, Nelson JK, Erwin S, O’Brien JM, Schwartz MW. Using species distribution models to predict new occurrences for rare plants. Divers and Distrib. 2009:15: 565-576. doi: https://doi.org/10.1111/j.1472-4642.2009.00567.x

*2.3	I find the conclusions in this study not fully supported by its results – the authors discussed management implications based on previous research and knowledge around the species, but the link between those discussions and the result in this study requires contextual and confirmatory knowledge of the species’ ecology. I strongly suggest including additional results, such as response curves and prediction maps, to further explain what precise environmental conditions are predicted to be favourable by the model.*

Response: We agree and thank the reviewer for their helpful feedback on the question of better supporting our conclusions. We have added a new figure that shows the response of the pathogen occurrence to isothermality and its interaction with rainfall seasonality and watering (see Fig. 3). We have included maps for all climatic predictors as supplementary material to better contextualize the climatic conditions that may promote the occurrence of Verticillium wilt. In addition, we have added text to the Discussion section on lines 299-303 and 306-312 to better clarify the link between the climatic conditions predicted by our model and the *V. dahliae* occurrence observed in Granada province.

Regarding the suggestion of including predictive maps, providing prediction maps of *V. dahliae* occurrence is challenging as the pathogen depends on the presence of its host, as well as the olive grove characteristics, i.e., watering type and plant material origin (Rodríguez et al. 2009, 2011). Unfortunately, we have only access to these data at the sampled locations, but not for the entire Granada province, limiting our ability to make large-scale predictions. Finally, it is worth to mention that our goal is beyond providing suitability maps for *V. dahliae* occurrence, but rather to explore what climatic factors affect its presence (as stated in comment 2.2), and to do that, we provide effect size and response curve plots.

References used in response:

Rodríguez E, García-Garrido JM, García PA, Campos M. Large-scale epidemiological study and spatial patterns of Verticillium wilt in olive orchards in southern Spain. Crop Prot. 2009;28: 46-52. doi: <https://doi.org/10.1016/j.cropro.2008.08.009>

Rodríguez E, García-Garrido JM, García PA, Campos M. Implications of plant material origin, land use history and soil properties in the incidence of Verticillium wilt in olive groves. J Plant Pathol. 2011;93(1): 111-118. doi: <http://dx.doi.org/10.4454/jpp.v93i1.280>

*Please find below a list of specific comments, in relation to line #

2.4	#44 this sentence can be shortened and merged with the previous*

Response: We agree. We have rephrased the sentence as follows:

“*V. dahliae* is of great concern in Mediterranean olive groves, where the olive species under cultivation (i.e., *Olea europaea* L. subsp. *europaea*) have crucial economic, social and environmental value, and therefore, the European Union’s agricultural policy is mainly aimed at protecting it”. See lines 41-44. *2.5	#86 considering the amount of available data per taxa, pathogenic species are not particularly under-represented in species distribution modelling, see e.g. 10.1016/j.fbr.2020.01.002 for a summary*

Response: We thank the reviewer’s feedback. Ireland and collaborators’ study refers specifically to climatic niche modeling for fungal plant pathogens. However, Hao et al. (2020) is also a very interesting article and we have included it as follows:

“In fact, fungal plant pathogens are particularly under-represented in climatic niche modeling studies [36], but see [37].” See lines 85-87.

Reference used in response:

Ireland KB, Kriticos DJ. Why are plant pathogens under-represented in eco-climatic niche modelling?. Int J Pest Manage. 2019;65(3): 207-216. doi: 418 <https://doi.org/10.1080/09670874.2018.1543910>

Hao T, Guillera-Arroita G, May TW, Lahoz-Monfort JJ, Elith J. Using Species Distribution Models For Fungi. Fungal Biol Rev. 2020;34(2):74-88. doi: https://doi.org/10.1016/j.fbr.2020.01.002

*2.6	#146-147 depending on the size of groves, within-grove climatic variations could be important and considered as a covariate*

Response: We thank the reviewer for this interesting comment. The area of 50% of the olive grove parcels in our study was less than 0.011 km^2^ (i.e., 50th percentile = 0.011 km^2^ ≈ 1 ha) and the area of 95% was smaller than 0.126 km^2^. In order to capture the potential within-parcels climate variability, high-resolution climate data are required, which are not available for Granada province. We have added text to the Conclusions section on lines 344-346 to emphasize that further studies at finer scales would be desirable to explore this and other questions related to the micro-climate conditions impact on the pathogen occurrence. However, we would like to highlight here that our study is the first looking at the climate factors of *V. dahliae* occurrence on a large scale, and therefore, our motivation is to provide initial insights that encourage future research to advance this research gap.

*2.7	#150 it appears that ‘covariate’ is used to refer to factor variables in this manuscript, I suggest using only ‘covariate’ or ‘variable’ to refer to all predictors, as commonly practiced by the species distribution modelling community*

Response: Thank you for the comment. Indeed, we used the “covariate” term to refer to both categorical (“watering” and “plant material origin”) and continuous (“area”) non-climatic variables as suggested by the reviewer. See for example lines 158-159 and Fig 2.

*2.8	#188-189 are the data partitions sampled with replacement? Why not a standard 10-fold CV (dividing data into 10 distinct blocks and using 9 to train and 1 to test)*

Response: No, the data partitions were not sampled with replacement. However, we agree with the reviewer and have rerun the models using the standard 10-fold cross-validation approach (see lines 194-196. Thus, we have split the data into 10 distinct blocks and iteratively used 9 blocks to train and 1 block to assess the models' performance. See the attached script and the updated version of S2 Table. Please, note that the overall performance has improved compared with the one achieved by sampling without replacement (see lines 235-238).

*2.9	#231 I think these supplementary figures help readers a lot – mapped rasters of all continuous predictors can also be included in the Appendix, to better contextualise the environment in the study area*

Response: We totally agree and provide such information in S2 Fig. See also line 149 in the main manuscript.
