# Supplementary material for: Climatic drivers of Verticillium dahliae occurrence in Mediterranean olive-growing areas of southern Spain
Source: PLoS One. 2020 Dec 30;15(12):e0232648. doi: 10.1371/journal.pone.0232648 (PMC7773261; doi:10.1371/journal.pone.0232648)
Supplement: Attachment — Submitted filename: Response to Reviewers (2).docx [file pone.0232648.s007.docx]

***General comments to reviewers and Academic Editor***

Dear Mr. Di Febbraro:

Thank you for the opportunity to revise our manuscript, “Climatic drivers of *Verticillium dahliae* occurrence in Mediterranean olive-growing areas of southern Spain” to *PLOS ONE*. The two reviewers are positive about our study and are satisfied with the responses to the recommendations that they arose in the first round. We agree with all the minor amendments suggested by Reviewer #2 and have updated our manuscript accordingly. In our response below, we have italicized the reviewers’ comments. We look forward to your final decision on the revised manuscript.

**REFEREE REPORT(S):**

**Reviewer: 1**

Comments to the Author

*I congratulate with the authors for the excellent work. All my comments have been successfully addressed.*

Response: Thank you very much.

**Reviewer: 2**

Dear authors,

*Thank you for the opportunity to re-review the manuscript titled ‘Climatic drivers of Verticillium dahliae occurrence in Mediterranean olive-growing areas of southern Spain’. I am satisfied with the authors’ responses to the recommendations I raised in the first round. I believe this manuscript is now robust and valuable, with only a few minor tweaks, it can make a nice addition to the journal.*

*Although the changes from the previous version are not substantial, I am convinced by the authors’ responses to my previous comments, which demonstrated their comprehensive knowledge of the study system and I can now better appreciate the reasonings behind their conclusions. Below I detail my updated views on the concerns I raised in my previous review.*

*Regarding trialling more complicated modelling method: I am impressed that the authors fitted BRTs with multiple sets of parameters, and tested model performance rigorously. I think this result (that GLMs beat BRTs in performance) is worth mentioning in the text, or at least in an appendix, because other model-focused readers may also be interested in whether different modelling methods are tried for this research problem.*

*Regarding choice of covariates: I am convinced by the authors’ response that the covariates tried here are most suited to the study. Although I am still wondering whether seasonal and/or monthly climate data should be tested, since the authors identified that warm and wet conditions are most imported to V.dahliae, so maybe the specific susceptible month(s) are worth focusing on.*

*Regarding better contextualising the favourable conditions to V.dahliae: I think the new appendix maps of predictors will suffice, and I agree with the authors that occurrence prediction maps are not needed for this study.*

Response: Thank you very much for these positive comments. We have mentioned the BRT vs. GLM comparison in the “Modeling approach” and “Results” sections of the new version of the manuscript as follows:

“We carried out a sensitivity analysis to assess how much models performance changes when using a different modeling technique. To do that, we compared the prediction capacity of our top-ranked logistic model (see Table 1) with boosted regression trees using a 10-fold cross-validation (see S1 Appendix for details).” See lines 200-203.

“The model comparison results showed no evidence of improving predictive performance by the BRT models compared to that obtained by the top-ranked binomial model, supporting the linear regression modeling approach adopted in our study (see S3 Table in S1 Appendix).” See lines 241-244.

In addition, we have included the mentioned comparison as an appendix along with the R code to replicate the analysis (see S1 Appendix). We have not included the results in the main manuscript because we think that comparing different modeling approaches is beyond the scope of our study.

We will definitely consider seasonal and monthly climatic variables in future studies on the *V. dahliae* drivers.

*I have a few other minor suggestions:*

*2.1	In table 1, I am not sure whether the hypothesis column is necessary, I believe the information is better conveyed in text, through a more detailed elaboration on how the top models supported the specific hypothesis.*

Response: We agree and have removed the hypotheses column from Table 1. The information is conveyed in the text (please see lines 169-173) and how the top models supported the specific hypotheses is shown in lines 206-209.

*2.2	Line 245: I suggest “compound” or “composite” over “sophisticated”, but I agree with the “primary” wording.*

Response: We agree and appreciate the comment. We have replaced “sophisticated” with “compound” throughout the manuscript. See for example lines 32 and 248.

*2.3	Line 286-290: I think the observed effect of plant nursery origin in the model deserves more discussion. Although it is beyond the scope of this study, it hints that non-climatic factors are also significant in moderating disease risk, this is worthwhile knowing from a management perspective.*

Response: We agree and have added the following paragraph in the new version of the manuscript. Please see lines 291-296.

“This result underlines the importance of abiotic factors in reducing disease risk during the early stages of cultivation. Infected planting material from the nursery is one of the main reasons for *V. dahliae* infestation in free-pathogen growing areas [33]. In order to prevent the pathogen from spreading, particularly the most virulent forms, such as the defoliating pathotype (D), farmers should use only pathogen-tested propagative material.”

Reference

Rodríguez E, García-Garrido JM, García PA, Campos M. Implications of plant material origin, land use history and soil properties in the incidence of Verticillium wilt in olive groves. J Plant Pathol. 2011;93(1): 111-118. doi: http://dx.doi.org/10.4454/jpp.v93i1.280

*2.4	Appendix S1: the colour scale on the last figure is confusing (it looks intuitively like a value that ranges from 0 to 1), I suggest changing it.*

Response: We agree. We have replaced the old palette with a more intuitive one. Please see S1 Fig.
